# Alzheimer’s Disease Prediction Using Attention Mechanism with Dual-Phase ^18^F-Florbetaben Images

**DOI:** 10.1007/s13139-022-00767-1

**Published:** 2022-08-12

**Authors:** Hyeon Kang, Do-Young Kang

**Affiliations:** 1 Institute of Convergence BioHealth, Dong-A University, Busan, Republic of Korea; 2 Department of Nuclear Medicine, Institute of Convergence Bio-Health, Dong-A University College of Medicine, 32, Daesingongwon-ro, Seo-gu, Busan, Republic of Korea; 3 Department of Translational Biomedical Sciences, Dong-A University, Busan, Republic of Korea

**Keywords:** Alzheimer’s disease, Amyloid-β, Blood perfusion, Functional neuroimaging, Machine learning, Neural network

## Abstract

**Introduction:**

Amyloid-beta (Aβ) imaging test plays an important role in the early diagnosis and research of biomarkers of Alzheimer’s disease (AD) but a single test may produce Aβ-negative AD or Aβ-positive cognitively normal (CN). In this study, we aimed to distinguish AD from CN with dual-phase ^18^F-Florbetaben (FBB) via a deep learning–based attention method and evaluate the AD positivity scores compared to late-phase FBB which is currently adopted for AD diagnosis.

**Materials and Methods:**

A total of 264 patients (74 CN and 190 AD), who underwent FBB imaging test and neuropsychological tests, were retrospectively analyzed. Early- and delay-phase FBB images were spatially normalized with an in-house FBB template. The regional standard uptake value ratios were calculated with the cerebellar region as a reference region and used as independent variables that predict the diagnostic label assigned to the raw image.

**Results:**

AD positivity scores estimated from dual-phase FBB showed better accuracy (ACC) and area under the receiver operating characteristic curve (AUROC) for AD detection (ACC: 0.858, AUROC: 0.831) than those from delay phase FBB imaging (ACC: 0.821, AUROC: 0.794). AD positivity score estimated by dual-phase FBB (R: −0.5412) shows a higher correlation with psychological test compared to only dFBB (R: −0.2975). In the relevance analysis, we observed that LSTM uses different time and regions of early-phase FBB for each disease group for AD detection.

**Conclusions:**

These results show that the aggregated model with dual-phase FBB with long short-term memory and attention mechanism can be used to provide a more accurate AD positivity score, which shows a closer association with AD, than the prediction with only a single phase FBB.

## Introduction

Approximately 50 million people worldwide suffer from dementia, and nearly 10 million new cases occur every year. The total population with such dementia is expected to be 82 million by 2030 and 152 million by 2050 [1]. Alzheimer’s disease (AD), the most common cause of dementia, is complex and multi-factorial in elucidating the continuum of conditions leading to asymptomatic, mild cognitive impairment, and dementia. Amyloid-β (Aβ), which can be measured through positron emission tomography (PET) scan or cerebrospinal fluid analysis, is one of those defining the pathology of AD and is known as the earliest sign among AD biomarkers. Therefore, Aβ-related biomarkers have been studied for a clinical diagnostic index as well as for early diagnosis or prediction [2–4]. However, as AD is known to be affected by neurofibrillary tangles aggregated by hyperphosphorylated tau protein, genetics, and environmental influences as well [5], both Aβ-negative AD and Aβ-positive CN inevitably exist [6]. In addition, it is difficult to monitor the patient’s condition because Aβ plaques are already saturated by the time cognitive function clinically declines [7]. These facts remind us how additional AD biomarkers are required to understand and respond to AD. 18F-Fluorodeoxyglucose (FDG), which is a radiopharmaceutical that enables imaging of changes in glucose metabolism in brain tissue, is another one of representative AD biomarker. Hypometabolism, which is measured using FDG-PET, is known to be associated with neurodegeneration and cognitive decline [8]. However, such a series of PET imaging tests have drawbacks that make patients who need a diagnosis or longitudinal studies for AD undergo relatively frequent radiation exposure and high financial expenditure.

Aβ uptake in early-phase Aβ-PET is known to be a potential perfusion imaging modality that reflects cerebral blood flow [9–11]. Reference [4] reviewed the coupled relationship between hypoperfusion which causes deleterious changes in neurons and cerebral hypometabolism which underlies neuronal/synaptic dysfunction with the respective associations with cognitive impairment. Given an adequate evaluation of neuronal function and Aβ load from dual-phase Aβ-PET imaging, we may be able to provide patients with a more accurate AD diagnosis and prognostic evaluation without compromising patient convenience. Compared to late-phase Aβ-PET, however, there is no consensus or a well-established guide regarding how to interpret and evaluate the potential perfusion imaging for AD.

In the field of imaging biomarkers, various efforts have been made to provide an improved quality of medical services continuously. In particular, the latest technologies incorporating artificial intelligence have been reported to show a consistent inference and classification performance comparable to a human doctor. Such technologies are excellent at not only reducing a portion of manual labor of human doctors but also addressing inter-observation problems [12, 13]. In addition, machine learning–based studies on imaging for AD biomarkers are also actively reported [14]. Existing machine learning–based studies for AD have commonly suggested some predictive models that learn single or more than two kinds of imaging data such as magnetic resonance imaging, FDG, or Aβ-PET. Those attempts using a variety of information for AD detection could be appropriate solutions that address the complex and heterogeneous characteristics of AD.

In this study, we aimed to develop and evaluate an improved AD prediction model in the machine learning algorithm by engaging with dynamic early-phase Aβ-PET as well as single late-phase Aβ-PET conventionally used for AD diagnosis. The method included (1) extracting the mean of the standard uptake value ratio (SUVr) with a consistent area from individual dual-phase Aβ-PET imaging; (2) selecting a machine learning–based predictive model, which estimates the AD positivity score; and (3) comparing the classification performance among models and evaluating the association between predicted AD positivity scores and cognitive function or occurrence of AD.

## Materials and Methods

### Participants

We adopted FBB PET as an imaging biomarker to evaluate Aβ and retrospectively recruited subjects who visited the Department of Neurology and Nuclear Medicine of the Dong-A University Hospital (DAUH) and underwent dual-phase FBB from November 2015 to June 2020. The total number of subjects was 264, consisting of 74 cognitive normal (CN) and 190 AD. Detailed demographic data of the participants are presented in Table 1. All CN cases had normal age-, gender-, and education-adjusted performance on standardized cognitive tests. The AD participants met the following inclusion criteria: (1) criteria for dementia according to the Diagnostic and Statistical Manual of Mental Disorders 4th Edition (DSM-IV-TR) [15] and (2) the criteria for probable AD according to the NIA-AA core clinical criteria [16]. The individual FBB PET imaging for Aβ load was visually evaluated by the brain Aβ plaque load (BAPL) scoring system, which defines a BAPL score of 1 (no Aβ load), 2 (minor Aβ load), and 3 (significant Aβ load) [17]. Dong-A University Hospital Institutional Review Board (DAUHIRB) reviewed this study with the member who participated in Institutional Review Board Membership List III and finally approved this study protocol (DAUHIRB-17-108). All procedures for data acquisition were by the ethical standards of DAUHIRB with the 1964 Helsinki Declaration and its later amendments or comparable ethical standards. We guarantee that informed consent was obtained from all participants for this study.

**Table 1:** Demographics of experimental data with dual-phase F18-Florbetaben imaging

Variables	CN	AD	Total	*p*-value
#	74	190	264	N/A
*Sex (F/M)*	49/25	102/88	151/113	0.065
*Age*	70.24 ± 7.41	71.95 ± 8.84	71.47 ± 8.48	0.142
*FBB reading* *(Aβ (−)/Aβ (+))*	59/15	32/158	91/173	< 0.001
Education (y)	9.30 ± 4.09	10.10 ± 4.48	9.88 ± 4.39	0.188
*K-MMSE*	27.50 ± 1.71	19.52 ± 4.25	21.62 ± 5.14	< 0.001

*CN*, cognitively normal; *AD*, Alzheimer’s disease; *K-MMSE*, Korean version of Mini Mental State Examination

### PET Acquisition

All FBB PET imaging was performed using a Biograph 40mCT Flow PET/CT scanner (Siemens Healthcare, Knoxville, TN, USA) and reconstructed through UltraHD-PET (TrueX-TOF). A dose of 300 MBq FBB was injected intravenously in resting conditions. Dynamic frames were acquired from 0 to 20 min and from 90 to 120 min post-injection after helical CT with a 0.5-s rotation time at 100 kVp and 228 mAs. The image acquisition time for dual-phase FBB PET was determined by related studies to sufficiently include the peak of Aβ uptake for early-phase FBB PET (eFBB) and the manufacturer’s recommendations for delay-phase FBB PET (dFBB) [10, 17, 18]. The acquired dynamic eFBB and static dFBB were 27 frames of 128 × 128 × 110 (3.19 mm × 3.19 mm × 1.5 mm) resliced from a field of view of 408 mm × 408 mm × 165 mm, and one frame of 400 × 400 × 110 (1.02 mm × 1.02 mm × 1.5 mm) resliced from a field of view of 408 mm × 408 mm × 165 mm, respectively. Static eFBB to evaluate potential perfusion was made by averaging the frames corresponding to 2–7 mins from dynamic eFBB. The optimal time period required to obtain static eFBB was internally determined using the approach in Reference [10].

### Data Pre-processing

We adopted a series of pre-processing procedures to extract regional mean SUVr for dynamic eFBB or static eFBB/dFBB, respectively, and each step was as follows. For the spatial normalization of all PET images, we used an in-house eFBB PET template [19], which averaged 8 CN and 8 AD randomly selected from the spatially normalized FBB data pool in Montreal Neurological Institute (MNI) space [20]. Each static eFBB was spatially non-linearly registered to the template space. For the dynamic eFBB of a case, we created a deformation field to represent the transformation from the mean of the total number of frames to the template space and applied it to each frame. The deformation field for a dFBB was identical to that derived from spatial normalization of the matched static eFBB [21]. As a result, the spatially registered imaging was in a voxel space of 95 × 79 × 68 (height × width × depth). We merged the Hammers atlas [22] into 7 representative regions (frontal lobe, temporal lobe, parietal lobe, anterior cingulate cortex, posterior cingulate cortex, and cerebellum) for the reference region for count normalization and volume of interest for estimating the mean SUVr. After spatial normalization, the intensities of each image were normalized with respect to the mean uptake of the whole cerebellar region as a reference region. Finally, for static eFBB/dFBB and dynamic eFBB, regional mean SUVr of 6 × 1 and regional time-activity curve (TAC) data of 6 × 27 (number of target regions × temporal length) were obtained, respectively.

### Calculation of AD Positivity Score Based on Brain Blood Perfusion and Amyloid-β Plaque

**Fig. 1:**
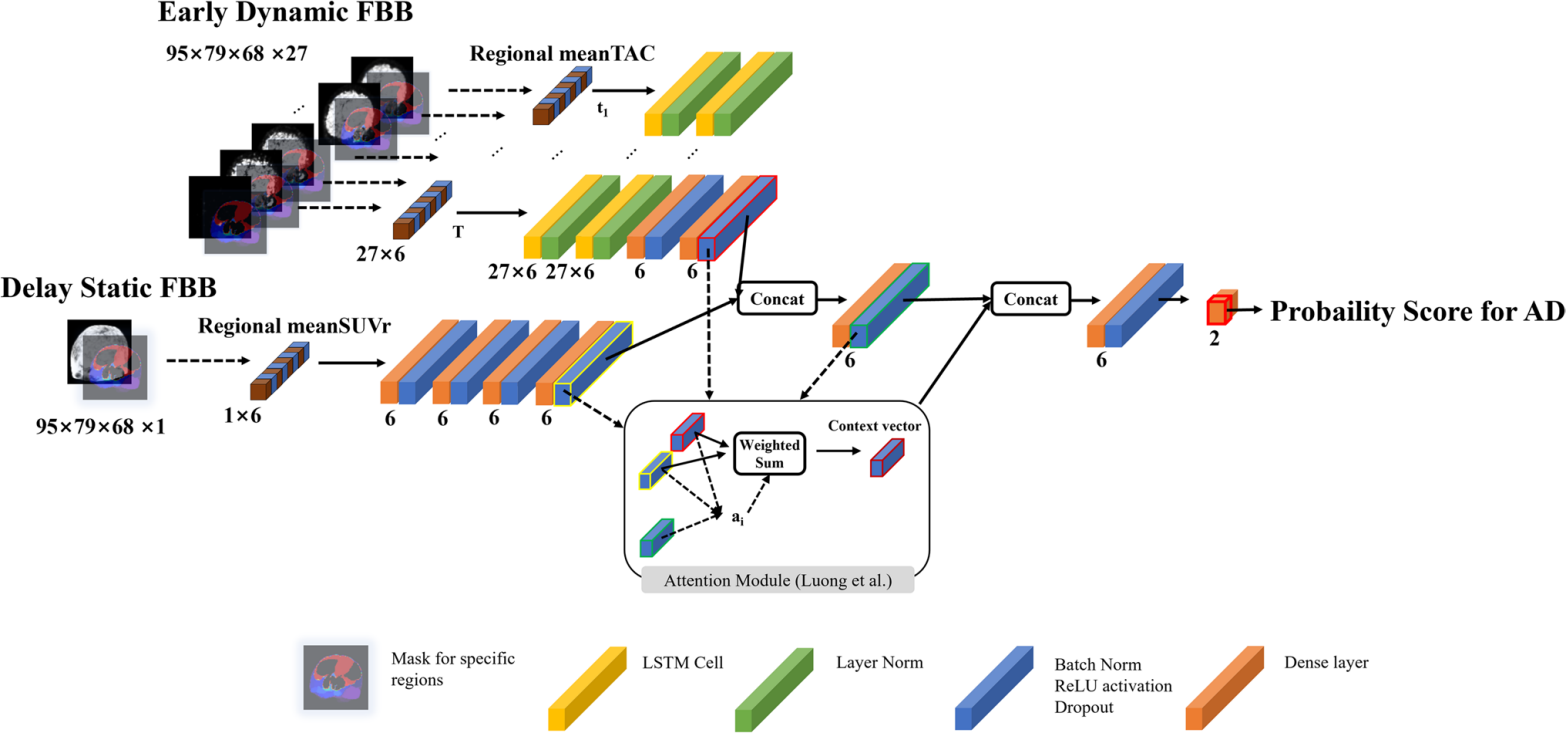
Overview of the proposed deep learning–based framework to estimate AD positivity score with dual-phase 18F-Florbetaben (FBB) PET imaging. For a given dual-phase FBB, we extracted regional meanTAC and SUVr data after pre-processing step for both phases of FBB and feed them into LSTM and simple dense layer, respectively, to obtain the phase-specific feature vectors. Those features are aggregated with concatenation and followed dense layer. The phase-specific features and 1st aggregated feature are used to make context vector using attention mechanism. Finally, the context vector and 1st aggregated features are pooled with concatenation and used to infer AD positivity score

To calculate the AD positivity score from regional SUVr, we build a neural network (NN)–based classification model to predict the probability of whether the given regional TAC or mean SUVr data belong to the CN or AD distribution. Figure 1 shows the structure of our proposed framework that predicts AD using dual-phase FBB. The whole aggregated NN (NN_aggregated_) in Fig. 1 consists of three modular networks: long short-term memory (LSTM_eFBB_) model to extract temporal features from dynamic eFBB, feedforward neural network (NN_dFBB_) for dFBB, and following NN (NN_Dx_) to make a final diagnosis decision from the phase-specific features for each phase of FBB delivered from the preceding layers. In particular, we adopted an attention mechanism [23] to adaptively select the phase-specific features for AD detection under biomarkers’ disagreement. We describe the details of each modular networks and the attention mechanism layer connecting them in the following section.

Context vector encoded by attention mechanism layer and 1st aggregated features are pooled and used to infer AD positivity score. NN_Dx_ has an output layer with two nodes leading to the softmax function to interpret the model output as the probability for diagnostic labels, and their model parameters were trained to minimize the cross-entropy loss between the predicted probability and one-hot encoded actual label. To evaluate the efficacy and feasibility of the proposed model, we compared it against representative methods such as support vector machine (SVM) [24], and random forest (RF) [25] as a baseline.

### Three Modular Networks for Independent Feature Extraction and Aggregation

We built the whole network into a combination of individual modules that are responsible for the independent task of performing AD classification. Long short-term memory (LSTM) is well known for handling long-term dependencies of temporal features using three types of gates (input, forget, and output gates) and memory cells [26, 27]. LSTM_eFBB_ produces phase-specific features for AD classification from regional TAC data. We first applied this LSTM layer on regional TAC data (6 × 27) to produce the temporal feature. Then, we applied layer normalization to reduce training time and stabilizing the hidden state dynamics in the previous recurrent neural network layer [28]. All of LSTM layers in NN_aggregated_ were followed by individual layer normalization. After two layers of LSTM, we applied feed forward layer (FC) on the output (6 × 1) at the last time step to encode high-level phase-specific feature. All of FC in NN_aggregated_ were followed by the pre-defined layer block, which are batch normalization, ReLU activation [29], and dropout layer [30]. To encode phase-specific feature for dFBB, we used a 4-layer FC followed by the pre-defined layer block which was explained above. Finally, we produced comprehensive functional features from two types of phase-specific features and phase attention which we present in the following section and AD positivity score by applying single-layer FC (NN_Dx_).

### Attention Mechanism for Adaptive Phase-Specific Feature Selection

In this work, we focus on adaptive phase-specific feature selection to address biomarkers’ disagreement. We adopt an attention method proposed by Luong et al. [23] to adaptively select proper evidences to predict AD positivity. Assume that a subject has *N* phase-specific hidden features *h*_*i*_ with *i* ∈ [1, *P*], *H* ∈ *R*^*D*^ and *h*^′^ *with H*^′^ ∈ *R*^*D*^ as a 1st aggregated hidden feature by concatenating *N* phase-specific hidden features and applying single-layer FC (Fig. 1). To highlight more informative phase to form the 1st aggregated feature for AD detection, we introduce a phase context vector C created from *h*_*i*_, *h*^′^ as the input of this mechanism as follows:

1 $${e}_i=f\left({h}_i,\kern0.5em {h}^{\prime}\right),$$

2 $${a}_i=\frac{\exp \left({e}_i\right)}{\sum_{k=1}^N\exp \left({e}_k\right)},$$

3 $$C=\sum\nolimits_{i=1}^N{a}_i{h}_i$$

where *f* is simple neural network that aggregates all of phase-specific hidden features *h*_*i*_ and reference feature *h*^′^. The simple network can be written as follows:

4 $$A= softmax\left(\mathit{\tanh}\left( XW+b\right)\right)$$

Here, *X* is concatenated feature according to each phase between *h*_*i*_ and *h*^′^ as *X* ∈ *R*^*N* × 2*D*^. *W* and *b* are model parameters which will be learned to make attention score A with *W* ∈ *R*^2*D* × 1^, *b* ∈ *R*^*N* × 1^, *and A* ∈ *R*^*N* × 1^. Finally, phase context vector C is the weighted sum of H with A as (3). And the context vector C and 1st aggregated feature will be used to encode 2nd aggregated feature in Fig. 1.

### Detailed Parameters for Model Selection and Model Evaluation

For our experiment, we focused on showing that the model with dual-phase FBB is more useful for estimating AD positivity than a model with only dFBB. Therefore, we tried to simplify and unify the model structure and detailed parameters of each model as much as possible. NN-based models, including LSTM, have two hidden layers, with six nodes of each hidden layer. To prevent neural networks from overfitting, we apply L2 regularization with a weight of 0.01 and dropout layer with dropout rate of 0.2. The learning curves of all models were set to be trained up to 10,000 epochs but were stopped if the validation loss was not updated more than 200 times. The learning rate was 0.00001, and the Adam optimizer [31] was used for each setting. If the validation loss was not updated more than 100 times at a point, 0.001 of the decay rate was applied to the learning rate of the point.

SVM used in the experiment used a linear kernel as a kernel function. A radial basis function or polynomial kernel was also tested in an internal experiment but no meaningful difference was observed, and a simpler model was finally adopted to prevent overfitting. RF was trained with a max depth of 2 and a number of estimators of 1000, and gini inpurity [32] was used to measure the quality of a split. The hyperparameters of both comparative models were heuristically determined.

For model selection and evaluation, our dataset was split into training, validation, and testing with ratios of 0.6, 0.1, and 0.3, respectively. We use stratified sampling so that the ratio of diagnostic labels according to Aβ load in each data was same. The data split was the same for each phase of the dataset and all experiments. The previously preprocessed TAC and SUVr datasets were last subjected to min-max normalization before being input to a predictive model after the split.

The software used in this experiment was the SPM12 library and MATLAB R2020a for the data pre-processing, including spatial normalization, and count normalization, for evaluating the pre-processed image with t-contrast, and for calculating regional mean SUVr based on the Hammers atlas [22]. Keras 2.2.4 library and Python 3.6.9 were used to select and evaluate a model for estimating AD positivity. The experimental tool was implemented and tested on Linux Ubuntu 16.04 LTS with an Intel Core i7-6800K CPU and two GPUs (NVIDIA GeForce GTX 1080).

### Statistical Analysis

We used independent-sample *t*-tests for numerical variables such as age and education and Pearson’s Chi-square test for categorical variables such as sex, FBB reading, and K-MMSE to determine whether the characteristics of subjects in our experimental dataset are biased according to the diagnostic label. For the demographic analysis, we used IBM SPSS statistics version 23. To evaluate the classification performance of trained models, we calculated the accuracy (ACC) and area under the receiver operating characteristic curve (AUROC) for AD detection using DeLong’s method [33] and Spearman correlation between predicted AD positivity scores and neuropsychological tests/actual diagnostic label. For these processes, we used MedCalc version 18.9.1 (MedCalc Software). In all tests, the statistical significance level was set at *p* < 0.001 with a two-sided test.

## Results

### Data Demographics

As Table 1 shows, there was no statistically significant difference between the CN and AD groups in age, sex, and education variables. The results of K-MMSE (which is the dominant variable in the diagnosis of AD and reflects cognitive function) and dFBB readings (which reflect a state of Aβ plaque load) showed statistically significant differences between groups. Therefore, the retrospective data used in the experiment differed only in the cognitive function and hallmark pathology that directly affect the diagnostic label, but no bias was observed in other factors. Our experimental data included 20.83% of Aβ-positive CN and 16.84% of Aβ-negative AD.

### Pre-processed Imaging Data for TAC and SUVr

**Fig. 2:**
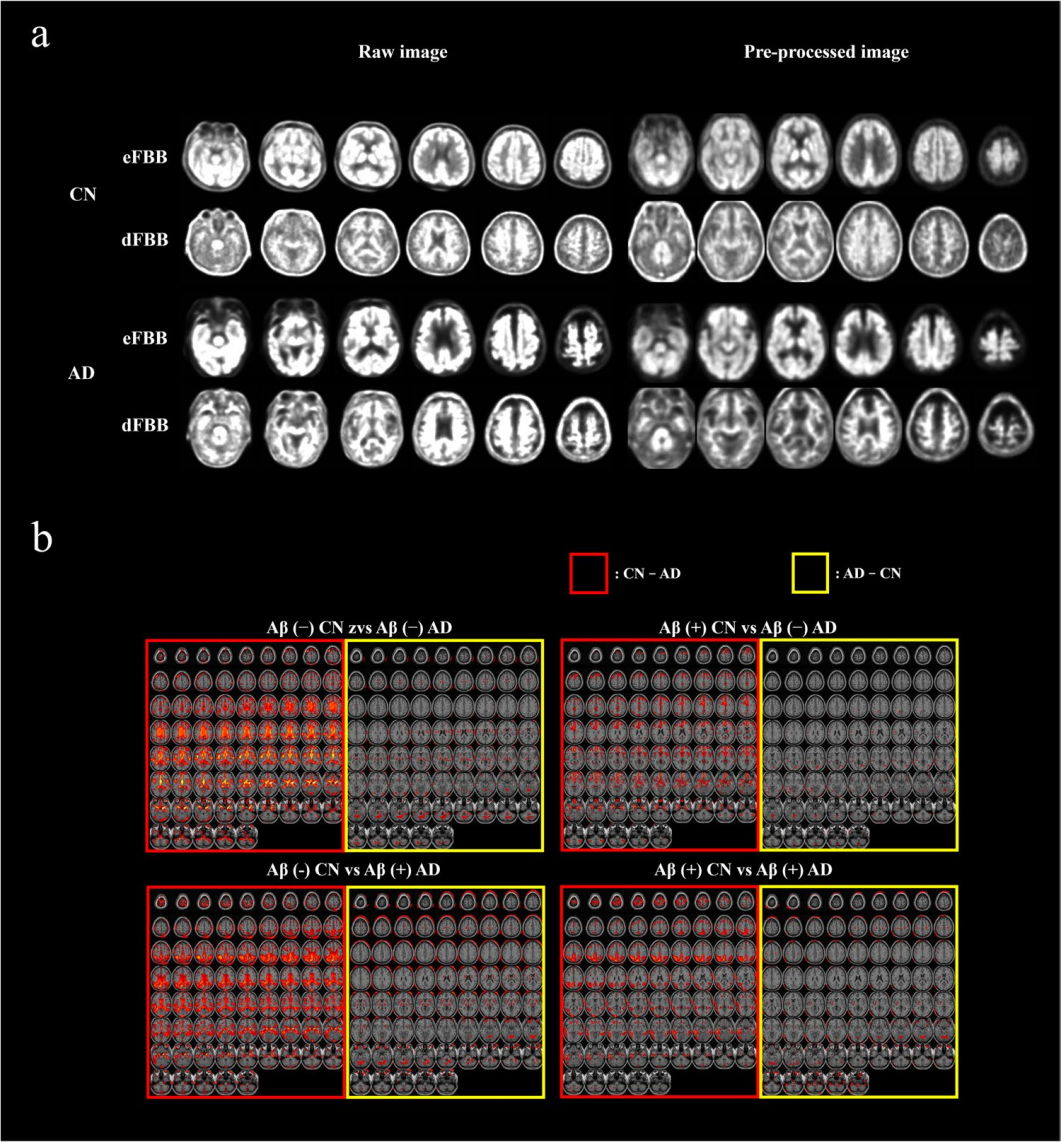
Pre-processed dual phase FBB PET (**a**) and t-contrast of early phase F^**18**^-Florbetaben PET according to Aβ distribution (**b**). The contrast was set to [1, −1] for cognitive normal vs. Alzheimer’s disease in SPM12

For the result of spatial registration, Fig. 2a shows static eFBB and dFBB registered in MNI space, which is randomly selected from each diagnostic label, compared with raw images of those in native space. As a result of pre-processing, it was confirmed that the spatial characteristics of individual imaging disappeared after they were transformed into MNI space but functional characteristics remained according to the diagnostic label.

In Fig. 2b, to check whether the functional information of eFBB on our pre-processing method and selected time period is feasible, eFBB (2–7 min) was observed by t-contrast according to the diagnostic label. The functional information of dFBB was omitted because the results have already been verified through previous studies [21]. In this study, t-contrast was applied to the eFBB images, and the voxel-wise difference between the two group (CN vs. AD) was calculated and visualized. As a result of t-contrast, the relative contrast of AD group is dominantly lower than CN, except for the cerebellar area in all of the 4 comparisons regardless of Aβ distribution.

### AD Classification Performance

Table 2 shows the AD classification performance of ML-based predictive models. LSTM (ACC: 0.792, AUROC: 0.775, F1: 0.849, G-mean: 0.773) was the best model for eFBB. RF (ACC: 0.736, AUROC: 0.584, F1: 0.835, G-mean: 0.467), NN (ACC: 0.726, AUROC: 0.648, F1: 0.813, G-mean: 0.467), and SVM (ACC: 0.708, AUROC: 0.746, F1: 0.763, G-mean: 0.740) followed. For the classifier of static dFBB, which is used for conventional FBB reading, NN (ACC: 0.821, AUROC: 0.794, F1: 0.872, G-mean: 0.792) was the best model for AD detection with dFBB (NN_dFBB_) and RF (ACC: 0.802, AUROC: 0.721, F1: 0.868, G-mean: 0.696) and SVM (ACC: 0.755, AUROC: 0.799, F1: 0.803, G-mean: 0.792) followed. In comparison among all kinds of FBB, the NN_aggregated_ was the best model (ACC: 0.858, AUROC: 0.831, F1: 0.901, G-mean: 0.828), which trained dual-phase FBB, followed by NN_dFBB_ that learned dFBB.

**Table 2:** Comparison of predictive performance for Alzheimer’s disease classification

Ac. phase	Model	Accuracy	AUROC	F1	G-mean
Early	SVM	0.708	0.746	0.763	0.740
	RF	0.736	0.584	0.835	0.467
	NN	0.726	0.648	0.813	0.622
	LSTM	0.792	0.775	0.849	0.773
Delay	SVM	0.755	0.799	0.803	0.792
	RF	0.802	0.721	0.868	0.696
	NN	0.821	0.794	0.872	0.792
Dual	LSTM+NN	0.858	0.831	0.901	0.828

*Ac. Phase* acquisition phase, *AUROC* area under receiver operating characteristic

**Fig. 3:**
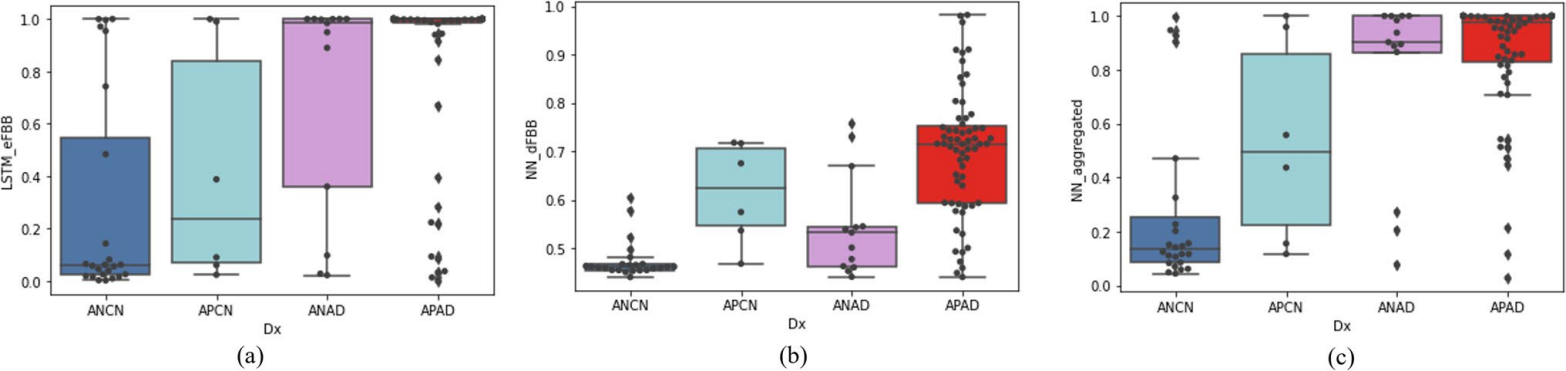
Predicted AD scores in Aβ-negative normal (Aβ (*−*) NC), Aβ-positive normal (Aβ (+) NC), Aβ-negative AD (Aβ (*−*) AD), Aβ-positive AD cases (Aβ (+) AD)

AD positivity scores measured by three models (NN_aggregated_, LSTM_eFBB_, and NN_dFBB_) with each phase of FBB (dual-phase FBB, dynamic eFBB, and static dFBB) in the test data are presented in Table 3. NN_aggregated_ (AUROC: 0.854) trained dual-phase FBB was able to detect AD better than LSTM_eFBB_ (AUROC: 0.841) and NN_dFBB_ (AUROC: 0.851). In comparison of AUROC in Aβ-negative distribution (Aβ (−) CN vs. Aβ (−) AD), the NN_aggregated_ (AUROC: 0.837) was the best, followed by LSTM_eFBB_ (AUROC: 0.792) and NN_dFBB_ (AUROC: 0.731). In Aβ-positive distribution (Aβ (+) CN vs. Aβ (+) AD), the NN_aggregated_ (AUROC: 0.901) was the best as well, followed by LSTM_eFBB_ (AUROC: 0.812) and NN_dFBB_ (AUROC: 0.706). Figure 3 shows the distribution of AD positivity scores predicted by the trained models on the test set. Figure 3(b) shows that there are many misclassifications in the distribution of Aβ (+) CN and Aβ (−) AD because NN_dFBB_ is only referring to the Aβ pathology. On the other hand, Fig. 3a and c demonstrate that LSTM_eFBB_ and NN_aggregated_ can relatively correctly predict the AD positivity score in the distribution of Aβ (+) CN and Aβ (−) AD. In particular, NN_aggregated_ using two features shows a remarkably correct classification of Amyloid negative AD than LSTM_eFBB_.

**Table 3:** Comparison of AUROC of AD positivity scores according to specific distribution

Pop./test (Ac. phase-model)	AUROC (SE)
Total	
Early FBB (LSTM_eFBB_)	0.841 (0.0417)
Delay FBB (NN_dFBB_)	0.851 (0.0394)
Dual FBB (LSTM+NN)	0.854 (0.0465)
Aβ (−) CN vs. Aβ (−) AD	
Early FBB (LSTM_eFBB_)	0.792 (0.0811)
Delay FBB (NN_dFBB_)	0.731 (0.1010)
Dual FBB (LSTM+NN)	0.837 (0.0729)
Aβ (+) CN vs. Aβ (+) AD	
Early FBB (LSTM_eFBB_)	0.812 (0.0846)
Delay FBB (NN_dFBB_)	0.706 (0.0964)
Dual FBB (NN_aggregated_)	0.901 (0.1570)

### Input and Feature Distribution According to the Visual Reading of dFBB and Diagnostic Label

**Fig. 4:**
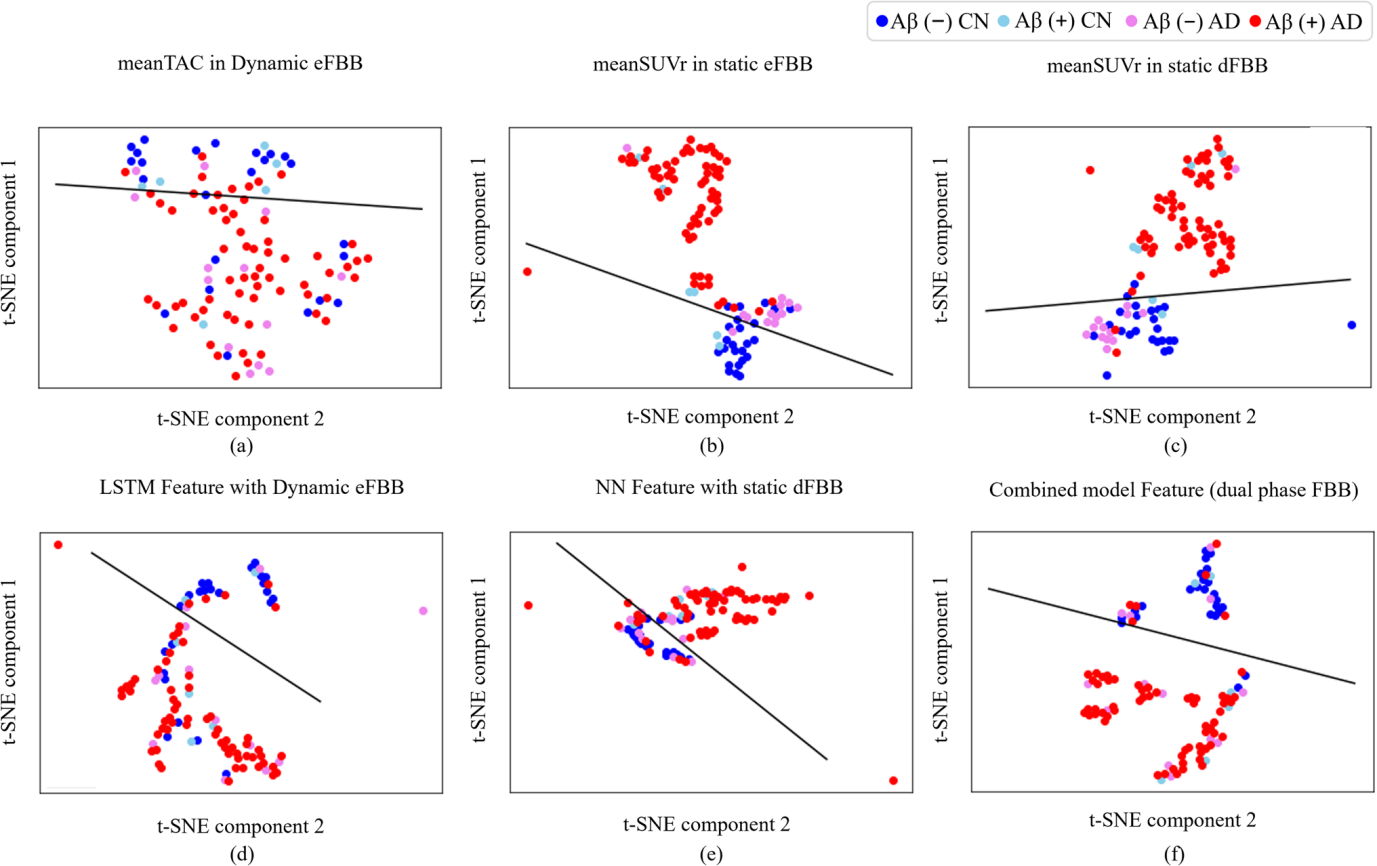
Distributions of model inputs and model features observed through t-SNE according to F^18^-Florbetaben reading label and diagnostic label. **a**–**c** show distributions of mean SUVr values used for a predictive model. **d**–**f** show distributions of feature vectors obtained from the last hidden layer of a neural network

Feature visualization provides a useful means of guessing how well a deep learning model understands the input data to achieve its learning goals. This can be addressed using t-distributed stochastic neighbor embedding (t-SNE), which is a kind of dimensionality reduction method designed to visualize high-dimensional data in a two- or three-dimensional map [34]. t-SNE prepares a neural network to understand target data distribution and is iteratively trained by gradient descent method so that the distance between data points low-dimensional data representation is similar to that in high-dimensional space. In Fig. 4, the distributions of inputs and features in the last hidden layer of the NN-based model according to the phase of FBB are shown in a two-dimensional space using t-SNE. Figure 4c and e show the distribution of mean SUVr and features extracted from dFBB, and those do not seem to fully explain Aβ-positive CN and Aβ-negative AD. The distribution of mean SUVr and features extracted from eFBB shown in Fig. 4a, b, and d appears to be that Aβ-negative AD distribution is closer to Aβ-positive AD distribution compared to those extracted from dFBB. However, it is observed that the Aβ-positive CN distribution is still close to that of Aβ-positive AD. On the other hand, in Fig. 4f, the feature distribution extracted from dual-phase FBB showed the separated representation rather than entangled for Aβ-negative CN and Aβ-positive AD.

### Association Between AD Positivity Score and Neuropsychological Test

**Fig. 5:**
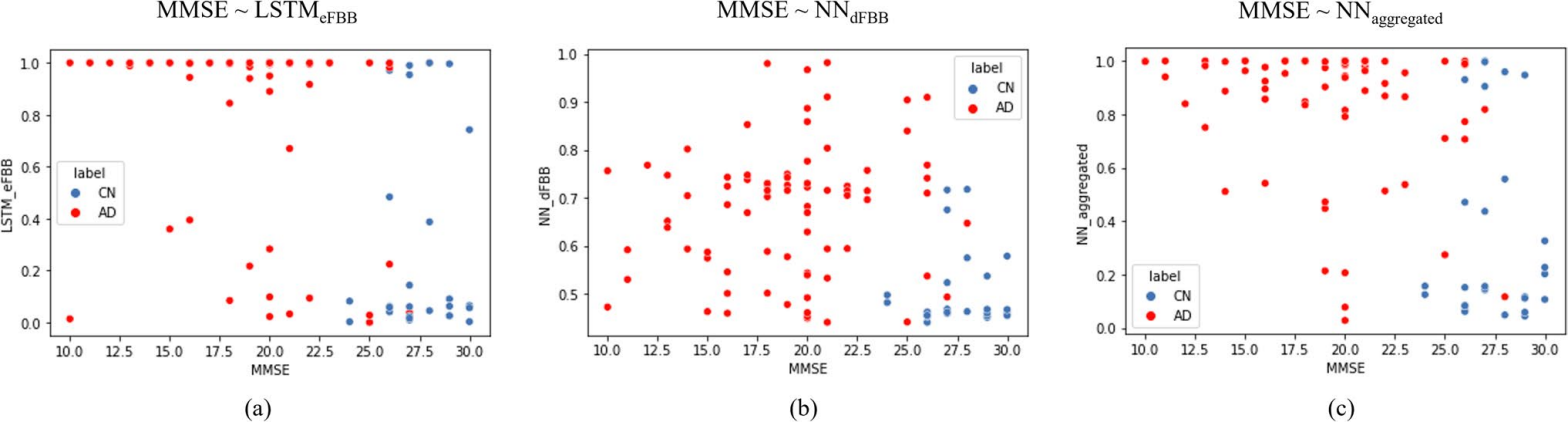
Association between predicted AD positivity scores from machine learning–based models and neuropsychological tests

Figure 5 shows the AD positivity score distribution of each phase of FBB according to neuropsychological test results. For AD cases with a low score of MMSE, NN_dFBB_ hardly shows a high AD positivity score. On the other hand, NN_aggregated_ and LSTM_eFBB_ suggested high AD positivity scores for cases with decreased cognitive function. In the correlation analysis, AD positivity score from NN_aggregated_ is best correlated with neuropsychological test results (R: −0.5412, *p* < 0.0001). The correlation of LSTM_eFBB_ (R: −0.4613, *p* < 0.0001) and NN_dFBB_ (R: −0.2975, *p* < 0.0022) followed.

### Observation of the Overall Behavior of the LSTM on Early-Phase FBB

**Fig. 6:**
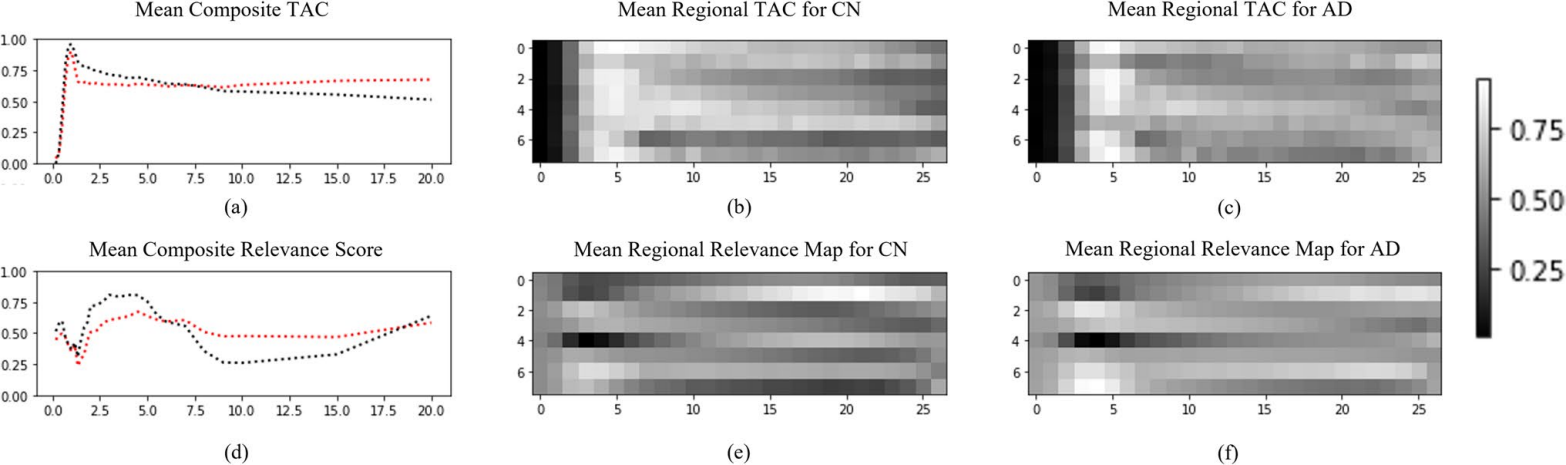
Regional time activity curve (TAC) learned by the LSTM to classify AD and the relevance map calculated from the model predicting the test set. In the second and third columns, the regional TAC with **b** and **c**, which were used for prediction by LSTM, and the relevance value applied to the model for the prediction explanation were visualized (relevance map) with **e** and **f**. **a** and **d** show the mean regional TAC along time axis (mean composite TAC) and mean regional relevance score along time axis (mean composite ) according to the diagnostic labels. The diagnostic labels include cognitive normal (CN, black) and Alzheimer’s disease (AD, red). We can observe that the distribution of relevance scores presenting the model behaviors as well as the TAC data is different between CN and AD population. In the relevance map, each column represents a regional SUVr at each time in the early phase FBB, and each row is an individual cortical area of the brain arranged in order of temporal lobe, anterior/posterior cingulate, frontal lobe, occipital lobe, and parietal lobe

Explaining a model prediction helps to understand the distribution of training data or the behaviors taken by the model to solve a given problem [35–37]. One approach for explaining deep NN decisions is by multiplying the partial derivative of the model prediction and the actual input feature, also referred to as simple Taylor decomposition [38], and this method also serves as a baseline for many related studies [39, 40]. The resulting relevance map can provide a feature-wise heatmap same as the input size and be understood as the product of sensitivity of how much the feature contributes to the model prediction and saliency of how much the feature is presented in the sample [40]. Figure 6 shows which part of the data the LSTM trained on eFBB observes for AD detection. In the comparison of the mean composite relevance in Fig. 6(b), CN shows a markedly high relevance in the 2nd to 5th frames and a remarkably low relevance in the 9th to 15th frames. On the other hand, AD shows a rather high relevance in the 4th to 7th frames and was generally maintained until the last frame. In the comparison of the mean regional relevance maps shown in € and (f), CN shows a remarkably high relevance in the anterior cingulate in the 15th to 25th frames. AD shows a higher overall relevance than CN, including the anterior cingulate and occipital lobe regions.

## Discussion

We designed a predictive model to successfully improve the conventional imaging biomarkers with only static dFBB by engaging in dynamic eFBB based on the following two assumptions: (1) The potential blood flow information included in eFBB is sufficiently distinguished from dFBB and they provide complementary information with respect to AD diagnosis. (2) The temporal information included in dynamic eFBB can be represented as an embedding vector representing blood flow information by the LSTM model. In the remaining paragraphs, we will elucidate the experimental results or related problems concerning the hypotheses above.

Compared with the use of only dFBB in the conventional context, to improve the accuracy of AD detection by engaging dual-phase FBB, eFBB and dFBB must contain sufficient complementary information regarding AD, that is, eFBB should be able to sufficiently explain AD in different aspects from dFBB. As shown in Fig. 2 and Table. 3, we tried to confirm whether the potential perfusion information of eFBB is suitable for this experiment. Even though the deformation field used for registration in eFBB was applied to dFBB, both eFBB and dFBB were located in the MNI space in our visual observation, and the Aβ load pattern in a region of gray matter was still observed in each preprocessed dFBB. In voxel-based analysis, hypo-perfusion was observed in the AD group regardless of the Aβ distribution (Fig. 2). From the comparison of characteristics between the same Aβ distributions in Table 3, it was observed that the AD positivity score from eFBB explained AD distribution better than from dFBB, which meant that it was difficult to discriminate the diagnostic label with dFBB in the same Aβ distribution. Therefore, the dynamic or static eFBB acquired from our experimental protocol is meant to be complementary to the uptake of dFBB for AD detection, and the improved classification performance of the NN_aggregated_ could be based on the additional potential blood flow data.

LSTM is a representative NN for time series data that ultimately understands the long-term contextual information by managing the cell state necessary to determine the output from the input over time through input, output, and forget gates [41]. In terms of research on medical data, LSTM has been frequently used in EEG/ECG [42], imaging reports, electronic health records, and static or dynamic imaging data [43, 44], which include temporal information. A common delay-phase static PET image is acquired at the acquisition time determined by investigating the pseudo-equilibrium interval in which specific binding remains stable through TAC data and considering other parameters such as image quality and diagnostic accuracy. In the case of the FBB radiotracer, the manufacturer provides acquisition time for the delay phase, not for the early phase. In eFBB, the optimal acquisition time interval closest to potential perfusion cannot be found in the stable state owing to the curve that changes rapidly around the peak; therefore, the interval must be determined exploratory. Even the interval for ideal potential perfusion imaging is not deterministic and may vary from case to case. As a related work, it was mainly considered in studies that explored a specific acquisition time based on the similarity between eFBB and FDG images. They randomly selected an interval, including the peak uptake[11], or searched for a combination of the start time and time window to determine the acquisition time with the correlation most similar to FDG [10]. Figure 6 shows that the temporal and spatial features observed by LSTM trained on the eFBB differ according to the diagnostic label. These results suggest the presence of the temporal features of the eFBB for AD detection and non-determinism of the acquisition time interval of the ideal potential perfusion image. In Table 2, the LSTM model showed better performance than the NN model trained static eFBB at 2–7 min, which had a good correlation with FDG in our prior study [18]. These experimental results may indicate that the LSTM could understand the temporal features required for AD classification from potential perfusion information in dynamic eFBB and the calculation of optimal acquisition time could be omitted.

Figure 3a and b show that eFBB and dFBB discriminate AD from CN using different features of each image. In Fig. 3b, most misclassifications occurred in the Aβ-positive CN and Aβ-negative AD populations, whereas, in Fig. 6a, the eFBB classifier consistently scores a proper AD positivity for CN or AD regardless of Aβ distribution. Therefore, it could be considered that the performance of the dual-phase FBB classifier originates from the state of neuronal injury by comprehensively evaluating the degree of hypo-perfusion from eFBB and Aβ plaque deposition from dFBB, respectively (Fig. 3c). In Table 3, AD positivity scores calculated by dual-phase FBB for the entire population showed the AUROC, which had no statistically significant difference compared to the MMSE, and better classification performance than those calculated using only dFBB regardless of Aβ distribution. These results may indicate that it is possible to improve the evaluation of the degree of neuronal damage in research or clinically when the AD positivity score of dual-phase FBB is provided. In addition, it could provide a quantitative index to nuclear medicine physicians to explain false negative/positive cases in FBB imaging tests. This quantitative method could be considered for application to other types of tracers or PET imaging where early-phase PET reflects potential perfusion information.

This study proposes a quantitative method for the interpretation of dual-phase FBB at this point when the evaluation criteria for potential perfusion information of eFBB have not yet been established. Ultimately, it could help to reduce the radiation exposure and costs for patients with AD, and for a nuclear medicine physician, it could be a helpful tool in visual assessment for dual-phase FBB. On the other hand, as a limitation of this study, the predictive model analyzing dual-phase FBB needs to be evaluated in terms of external validation or clinical validity in the future. As mentioned earlier, AD is associated with neurofibrillary tangles aggregated by phosphorylated tau, CSF biomarker, genetics, and environmental factors, in addition to Aβ plaque accumulation. Given additional clinical and laboratory data in the future, it would be possible to develop a predictive model that aggregates various predictive factors for AD in addition to improving the performance of the quantitative model in this study. Furthermore, if a suitable amount of data is collected for the study, the application of the CNN algorithm, which is recently playing an important role as an image processing method, is left for our future work.

## Conclusion

In this paper, we report on how to interpret dual-phase FBB using ML-based models and their evaluation results. In comparison with the AD classification, the model trained on mean SUVr extracted from dual-phase FBB imaging (ACC: 0.858, AUROC: 0.831) showed better AD classification than single-phase FBB, eFBB (ACC: 0.792, AUROC: 0.775), or dFBB (ACC: 0.821, AUROC: 0.794). In addition, the AD positivity score estimated by dual-phase FBB (R_MMSE_: −0.5412) shows a higher correlation with psychological test result compared to only dFBB (R_MMSE_: −0.2975). These experimental results show that the proposed method could be used to interpret eFBB in dual-phase FBB and that by reflecting eFBB into the current reading system, Aβ-PET reading, AD diagnosis, or the monitoring system could be improved.

## Data Availability

The data used for the study are available on request from the corresponding author.
